# MOFs-Derived Zn-Based Catalysts in Acetylene Acetoxylation

**DOI:** 10.3390/nano12010098

**Published:** 2021-12-29

**Authors:** Mengli Li, Zhuang Xu, Yuhao Chen, Guowang Shen, Xugen Wang, Bin Dai

**Affiliations:** 1 School of Chemistry and Chemical Engineering, Shihezi University, Shihezi 832000, China; 20192007095@stu.shzu.edu.cn (M.L.); 20182007103@stu.shzu.edu.cn (Z.X.); chenyuhao@stu.shzu.edu.cn (Y.C.); 20202107050@stu.shzu.edu.cn (G.S.); 2 Key Laboratory for Green Processing of Chemical Engineering of Xinjiang Bingtuan, Shihezi 832000, China

**Keywords:** metal–organic frameworks, heteroatoms, acetylene acetoxylation

## Abstract

Metal–organic frameworks (MOFs)-derived materials with a large specific surface area and rich pore structures are favorable for catalytic performance. In this work, MOFs are successfully prepared. Through pyrolysis of MOFs under nitrogen gas, zinc-based catalysts with different active sites for acetylene acetoxylation are obtained. The influence of the oxygen atom, nitrogen atom, and coexistence of oxygen and nitrogen atoms on the structure and catalytic performance of MOFs-derived catalysts was investigated. According to the results, the catalysts with different catalytic activity are Zn-O-C (33%), Zn-O/N-C (27%), and Zn-N-C (12%). From the measurements of X-ray photoelectron spectroscopy (XPS), it can be confirmed that the formation of different active sites affects the electron cloud density of zinc. The electron cloud density of zinc affects the ability to attract CH_3_COOH, which makes catalysts different in terms of catalytic activity.

## 1. Introduction

Vinyl acetate (VAc) is a significant chemical raw material globally, which is a colorless and flammable liquid with a sweet ether taste. VAc is mainly used to produce polyvinyl acetate (PVAc), polyvinyl alcohol (PVA), vinyl acetate-ethylene copolymer emulsion (VAE), ethylene-vinyl acetate resin (EVA), and other necessary downstream chemical raw materials, and it has also been widely used in coatings, adhesives, vinylon, and other fields [1,2,3]. At present, vinyl acetate is manufactured from petroleum ethylene and calcium carbide acetylene [4]. Given the fact that China features a coal-rich, oil-poor, and low-gas energy structure [5,6], calcium carbide acetylene is the primary source to produce vinyl acetate.

After continuous exploration and research by predecessors, it is found that the catalyst prepared with zinc as the active component and activated carbon as the carrier shows better catalytic performance in the production of vinyl acetate by the acetylene method [7,8,9]. Although it has been used in industry, there are still many shortcomings, such as a low conversion rate, easy agglomeration, deactivation of active components, and carbon deposition on catalysts. To deal with these problems, He et al. improved the activity and stability of the catalyst by doping bimetallic Zn-Ni [10] and Zn-Co [11]. Some researchers prepared V_2_O_5_-ZnO and Fe_2_O_3_-ZnO composite catalysts to extend the service life of the catalyst, but the results were not satisfactory [12,13]. There were also researchers who used nitrogen [14], oxygen [15], and boron [16] to modify activated carbon for changing the electron cloud density around zinc by changing the defect degree of the carrier, and then improving the reaction activity of the catalysts. It has been proven that many functional groups exist on the activated carbon support. However, little is known about how they affect the catalyst performance. Many scholars hold different views [15,17], rather than a uniform conclusion.

MOFs are crystalline nanoporous materials with a framework structure composed of transition metal ions and organic ligands [18], with the metal components dispersed one by one through organic ligands. On account of the large specific surface area, abundant pore structure, and surface defects of MOFs, MOFs have become a research hotspot in the fields of gas storage, gas adsorption, separation, sensors, drug controllable release, and catalytic reactions [19,20,21,22,23,24,25]. Because of their excellent chemical and thermal stability, MOFs are also used as a template or precursor for the preparation of porous carbon materials or metal composite materials [26]. By changing the types of organic ligands, catalysts doped with different elements can be prepared by simple calcination. Herein, we speculate that MOFs-derived materials can be used for catalyzing acetylene acetoxylation.

At present, the relationship between the active component and the carrier during the reaction process is not clear. This study mainly used MOFs as precursors of catalysts, after calcination under inert atmosphere; Zn-O-C, Zn-O/N-C, and Zn-N-C catalysts with different active sites were obtained, respectively; and the influence of different zinc coordination modes on the reaction was investigated.

## 2. Materials and Methods

### 2.1. Materials

Zinc nitrate hexahydrate (Zn(NO_3_)_2_·6H_2_O, 99.0%), terephthalic acid (H_2_BTC, 99.0%), N,N-dimethylformamide (DMF, 99.0%), dimethylimidazole (2-MI, 98.0%), methanol (99.5%), triethylenediamine (TEDA, 99.0%), and pyridine (PD, 99.5%), all reagents, were of analytical grade; no further purification was required.

### 2.2. Catalysts’ Preparation

#### 2.2.1. Preparation of Zn-O-C Catalyst Using MOF5 Precursor

MOF5 was prepared according to the method reported in the literature [27]. First, 2.23 g of Zn(NO_3_)_2_·6H_2_O and 0.67 g of H_2_BDC were dissolved in 100 mL DMF solution, respectively; after the raw materials had dissolved entirely, the solution containing Zn(NO_3_)_2_·6H_2_O was slowly poured into the solution containing H_2_BTC. After the mixing, the solution was transparent and then underwent centrifugal collection at a speed of 800 r/min, then the solutions were poured into a 200 mL poly tetra fluoroethylene (PTFE) hydrothermal reactor autoclave and kept at 140 °C for 24 h. After cooling to room temperature, the solution was centrifuged at 8000 r/min and washed by DMF three times. To remove the unreacted solvent molecules, the sample was treated with ethanol overnight, and then dried at 80 °C to obtain the MOF5 precursor.

Secondly, the prepared MOF5 was calcined at 450 °C for 4 h with a heating rate of 5 °C/min in a tube furnace under the nitrogen atmosphere. Then, the required catalyst could be obtained by natural cooling to room temperature and was named Zn-O-C.

#### 2.2.2. Preparation of Zn-O/N-C Catalyst Using TEDA-PD Precursor

The preparation method was based on the synthesis of Ni-MOF [28]. The preparation details are as follows: 2.38 g Zn(NO_3_)_2_·6H_2_O was dispersed in 100 mL DMF solution, and 1.33 g H_2_BTC and 0.6 g TEDA were dispersed in 100 mL DMF solution, after the complete dissolution, and the Zn(NO_3_)_2_·6H_2_O solution was slowly poured into the solution containing H_2_BDC and TEDA, and then stirred for 15 min. Next, a certain amount of PD slowly poured, and then stirred for 12 h in an oil bath at 100 °C. When it was cooled down to room temperature, the precipitate was collected by centrifugation and washed with DMF solvent three times, and subsequently it was dried under vacuum at 80 °C for 10 h; the precursor was obtained and named TEDA-PD. The catalyst obtained after 450 °C calcination was named Zn-O/N-C.

#### 2.2.3. Preparation of Zn-N-C Catalyst Using 2-Methylimidazole Zinc Salt (ZIF8) Precursor

ZIF8 was prepared by the method reported in the literature [29]. Here, 2.23 g and 2.46 g of Zn(NO_3_)_2_·6H_2_O and 2-MI, respectively, were added into 40 mL methanol solution; when they were completely dissolved, the Zn(NO_3_)_2_·6H_2_O solution was slowly poured into the 2-MI solution, and then stirred for 24 h at 500 r/min. The precipitate was collected by centrifugation at a speed of 10,000 r/min, washed with methanol three times, and then dried in a vacuum drying oven at 80 °C for 12 h. Finally, the sample was ground to a powder to obtain ZIF8. The catalyst obtained after 550 °C calcination was named Zn-N-C.

### 2.3. Catalysts Characterization

The scanning electron microscope (SEM, JEOL, JSM-6490LV, Tokyo, Japan) instrument was used to image the material. The Fourier transform infrared (FT-IR, Thermo Scientific, Nicolet IS10, Waltham, MA, USA) spectra was used to identify chemical species. The specific surface area, pore volume, and pore size of the sample were recorded by an automatic fast physical adsorption analyzer (BET, Micromeritics, ASAP 2460, GA, USA), degased at 150 °C for 5 h, and then analyzed under liquid nitrogen at −196 °C to test. X-ray photoelectron spectroscopy (XPS, Thermo Fisher Scientific, ESCAlAB 250Xi, Waltham, MA, USA) was used to analyze the chemical states of elements. The thermogravimetric (TG, NETZSCH, STA 449F3, Selb, Germany) was used to test the thermal stability of the samples. The temperature-programmed desorption (TPD, Micromeritics, ASAP 2720, Norcross, GA, USA) experiment was used to determine the gas adsorption capacity and adsorption strength of the catalysts. Inductively coupled plasma mass spectrometry (ICP, Agilent, ICPOES730, Santa Clara, CA, USA) was used to test the zinc content of the samples, which used argon as the carrier gas.

### 2.4. Catalyst Evaluation

A self-assembled fixed-bed reaction device was used for the activity test. A stainless steel pipe with an inner diameter of 10 mm was filled with quartz sand and quartz wool at the bottom of the reaction tube for paving, and a certain amount of catalyst was added. Reaction conditions: heating temperature was set at 220 °C, the gas hourly space velocity of C_2_H_2_ (GHSV) was 500 h^−1^ and the molar ratio of C_2_H_2_/CH_3_COOH was 1:2.45. Before the reaction started, CH_3_COOH was used to activate the catalysts for 0.5 h, and C_2_H_2_ was introduced to start the reaction. Then, samples were taken every 1 h, using GC-9A (Shimadzu, Tokyo, Japan) gas chromatography to analyze the content of the obtained product, and the chromatographic column model was a PEG/20000 (Zhonghuida, Dalian, China) packed column.

## 3. Results and Discussion

### 3.1. Catalytic Performance Evaluation

**Figure 1:**
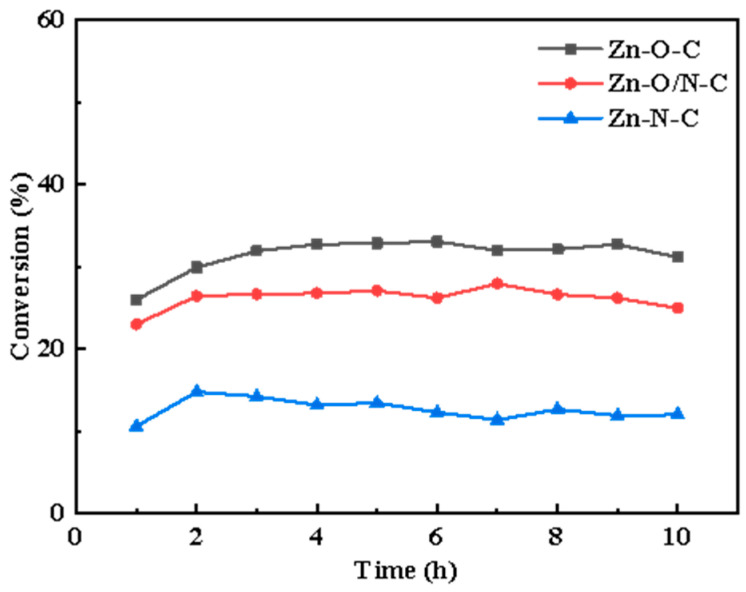
The conversion rate curves of Zn-O-C, Zn-O/N-C, and Zn-N-C catalysts over time, respectively.

Different active sites modes can be formed by doping different organic ligands. In this study, nine kinds of zinc-based MOF materials were prepared using organic ligands, including the following: 2-MI, H_2_BDC, H_2_BDC(NH_2_), H_2_BDC(NO_2_), TEDA, TETA (triethylenetetramine), and PD. The microscopic morphologies of eight kinds of materials are shown in Figure S1, and it can be observed that different structures such as petal-like, cube, cuboid, and sheet-like structures, respectively, were formed with the addition of different organic ligands. In a humid environment, water molecules will replace the organic ligands in MOFs, thereby destroying the crystal structure [30]; to remove the excess H_2_BDC, the organic matter in the precursor was volatilized and transformed into a loose porous structure, so we calcined MOFs to prepare catalysts. The conversion rate curves are shown in Figure S2, and it can be found that there is a certain difference in catalysts’ conversion. On this basis, we selected three kinds of materials with different zinc coordination modes, reactivity (Figure 1) and similar specific surface area (Table S1) to prepare Zn-O-C, Zn-O/N-C, and Zn-N-C catalysts with three-dimensional structures using MOF5, TEDA-PD, and ZIF8, respectively, as precursors.

**Figure 2:**
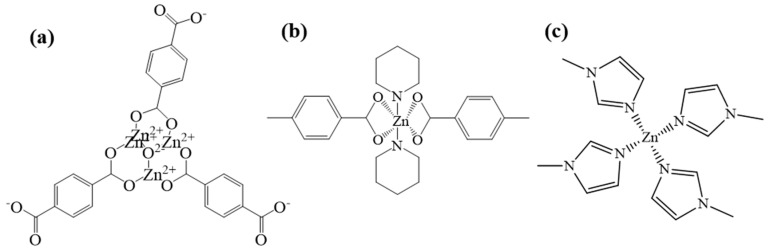
Schematic diagram of chemical structures for MOF5 (**a**), TEDA-PD (**b**), and ZIF8 (**c**).

MOF5 is one of the typical MOFs materials, and it uses zinc as the central metal and H_2_BDC as the organic ligand. First, four zinc atoms and an oxygen atom from H_2_BDC are connected to form the secondary structural unit Zn_4_O(BDC)_3_ [31] (Figure 2a); this second structural unit is in the shape of an octahedron, and metal ions are wrapped inside as the frame nodes. Because of its strong interaction force, the entire frame structure is more stable, and Zn_4_O(BDC)_3_ is connected with the ligand to form a three-dimensional skeleton with micropores. By adding other organic ligands, such as TEDA and PD, the coordination form of zinc can be changed into Zn-N and Zn-O, named as TEDA-PD. As shown in Figure 2b, TEDA-PD consists of a pair of zinc ions connected with oxygen atoms from four H_2_BTC in the horizontal direction and nitrogen atoms from TEDA in the vertical direction; the vertical connection was terminated using PD as an inhibitor and the thickness of the three-position frame can be controlled by adjusting the amount of PD added. In another case, in Figure 2c, ZIF8 possesses a zeolite imidazole ester skeleton structure, as an important type of MOFs materials; zinc acts as the central metal, while 2-MI acts as the organic ligand. Zinc is connected to four nitrogen atoms from 2-MI to form a three-dimensional cage structure. Both oxygen and nitrogen atoms have relatively strong electronegativity and, when they are coordinated with zinc, they will attract electrons around zinc. As different zinc coordination shows variation in the electron cloud density around zinc, this would affect the ability of the catalysts to attract CH_3_COOH, and finally lead to a different activity in the catalytic reaction.

**Figure 3:**
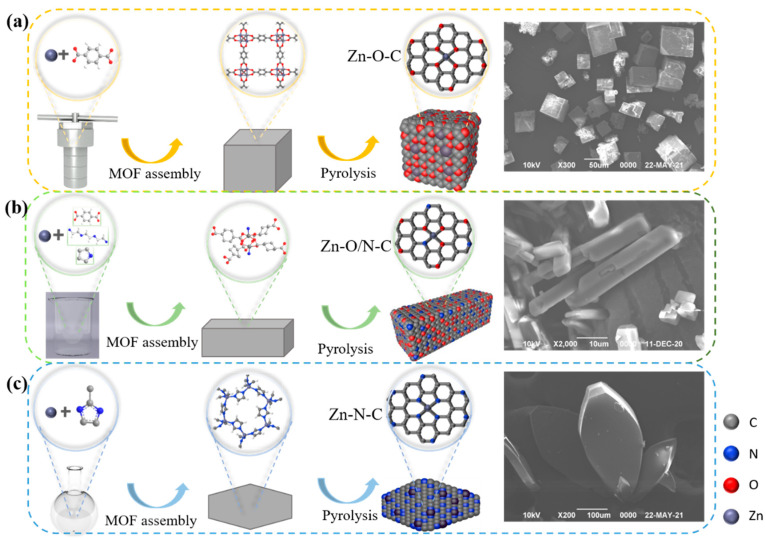
Schematic diagrams of the synthesis process and the SEM images of MOF5 (**a**), TEDA-PD (**b**), and ZIF8 (**c**), respectively.

Figure 3a–c shows the synthesis processes. The catalytic activity sequence is Zn-O-C (33%), Zn-O/N-C (27%), and Zn-N-C (12%); Zn-O-C shows better catalytic activity, and related characterizations are analyzed in the following section to explore the mechanisms for the different activities.

**Figure 4:**
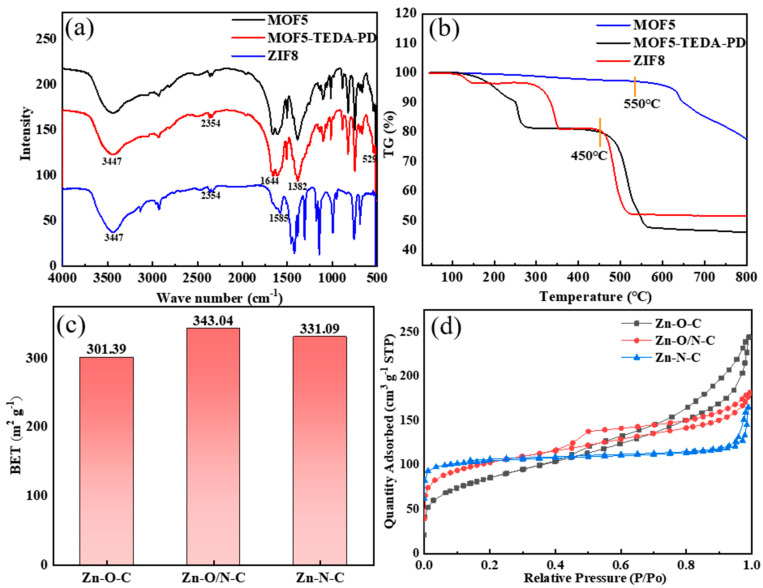
FT-IR spectra (**a**) and TG spectra (**b**) of MOF5, TEDA-PD, and ZIF8. Specific surface areas (**c**) and adsorption and desorption curves (**d**) of MOF5, TEDA-PD, and ZIF8 after calcination.

The infrared spectra of MOF5 and ZIF8 in Figure 4a are consistent with that of the reported literature [32,33], indicating that MOF5 and ZIF8 were successfully prepared. Among them, the absorption peak at 3447 cm^−1^ belongs to the O-H vibration, which was caused by physical adsorption of moisture in the atmosphere. The two sharp peaks around 2354 cm^−1^ can be attributed to C=O vibration, which came from the absorption of CO_2_ in the atmosphere. The peaks at 1644 cm^−1^ and 1382 cm^−1^ in MOF5 can be assigned to the symmetrical and asymmetrical stretching vibrations of O-C=O bond of the carboxyl group in H_2_BDC. The absorption peak of Zn-O bond in Zn_4_O tetrahedron is located at 529 cm^−1^. For the stretching vibration of the C=N bond at 1585 cm^−1^ in ZIF8, the band in the range of 500–1500 cm^−1^ is mainly caused by the bending vibration and stretching vibration in the imidazole ring.

According to the thermogravimetric spectra in Figure 4b, MOF5 began to collapse at about 500 °C, so 450 °C was chosen for calcination to avoid the collapse of MOFs. ZIF8 shows better thermal stability, which starts to decompose at about 600 °C; the specific surface area of the ZIF8 obtained by calcination at 450 °C was 11.77 m^2^/g. In comparison, ZIF8 was prepared at 550 °C to obtain similar specific surface areas to that of two other samples. MOF5, TEDA-PD, and ZIF8 were calcined to form catalysts rich in Zn-O-C, Zn-O/N-C, and Zn-N-C bonds, respectively; the BET (obtained by the Brunauer-Emmer-Teller algorithm) specific surface areas do not show much differences, with values of 301.40, 343.04, and 331.10 m^2^/g (Figure 4c), respectively. The adsorption and desorption curves (Figure 4d) show obvious hysteresis loops in all samples, indicating the existence of both micropores and mesopores.

**Figure 5:**
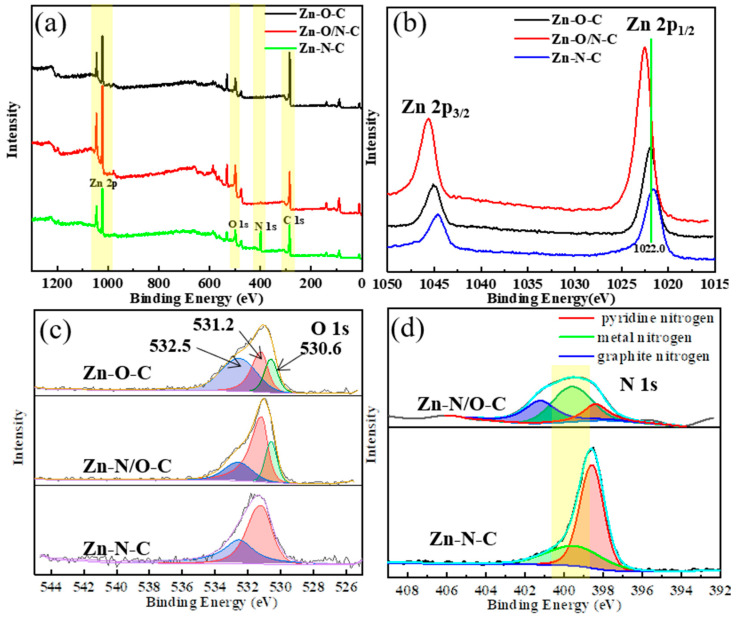
XPS pattern (**a**) and high-resolution XPS spectra of Zn 2p (**b**), O 1s (**c**), and N 1s (**d**) of Zn-O-C, Zn-O/N-C, and Zn-N-C.

In order to further verify the different coordination forms of zinc in different catalysts, we carried out an XPS test. From the XPS spectra in Figure 5a, it can be seen that the three materials all contain zinc, oxygen, and carbon. It is difficult to observe the presence of nitrogen in Zn-O/N-C, implying that Zn-O/N-C mainly consists of Zn-O-based coordination as well as a few Zn-N bonds; the element contents of each catalyst are shown in Figure S3a. Figure 5b–d displays high-resolution spectra of Zn 2p, O 1s, and N 1s of Zn-O-C, Zn-O/N-C, and Zn-N-C catalysts. In Figure 5b, the binding energies of Zn 2p_1/2_ and Zn 2p_3/2_ for Zn-O-C [34], Zn-N-C [35], and Zn-O/N-C are 1021.9 eV and 1045.0 eV, 1021.7 eV and 1044.6 eV, and 1022.0 eV and 1045.1 eV, respectively, indicating the existence of Zn^2+^ in the catalysts. Moreover, the values of binding energy positions for three catalysts are Zn-O/N-C > Zn-O-C > Zn-N-C, showing that the electron cloud density around zinc for Zn-O/N-C is the largest, while it is the smallest for Zn-N-C. The coordination state of the ligands around the zinc atoms is different [36], which is caused by the difference in the ability of oxygen and nitrogen atoms [37] to attract electrons. The electronegativity of oxygen atom is greater than that of nitrogen, which makes more electrons in Zn-O-C bias towards oxygen and leads to a reduction in the electron cloud density around zinc. It can be found that the binding energy position of Zn 2p moves to the high field direction. The electron cloud density around zinc of Zn-O/N-C is large, because zinc is connected to four oxygen atoms in the horizontal direction, while having more nitrogen connections in the vertical direction than Zn-O-C, so the peak for Zn 2p shifts to a higher binding energy. The shifts in the Zn 2p binding energy positions of the three catalysts indicate that zinc has different coordination modes. The O 1s pattern in Figure 5c shows that the binding energy value of Zn-O-C is the smallest. O 1s can be fitted into three peaks at 530.6 eV, 531.2 eV, and 532.5 eV. The first peak at 530.6 eV refers to the oxygen ion from the Zn-O bond in the lattice of ZnO [37], leading to zinc atoms existing in the form Zn-O [38]. The second peak at 531.2 eV is associated with the form of OH existing on the catalysts [39]. The third peak at 532.5 eV is related to the chemisorbed oxygen on the catalysts [40]. This implies that the three catalysts have different forms of oxygen presence. Compared with Figure S3b, in which C 1s is basically indistinguishable, this indicates that carbon atom does not affect the bonding of zinc. Next, the N 1s peak splitting of Zn-N-C and Zn-O/N-C catalysts is presented in Figure 5d; the N 1s peak of Zn-N-C can be fitted into two sub-peaks, located at 398.6 eV and 399.5 eV, representing pyridine nitrogen and metal nitrogen, respectively. Compared with the Zn-N-C catalyst, the nitrogen content in Zn-O/N-C is much smaller, showing that the catalyst is mainly coordinated by Zn-O bonds, and part of the metal nitrogen was replaced by graphite nitrogen (400.9 eV) [41], so the content of metal nitrogen reduced accordingly.

**Figure 6:**
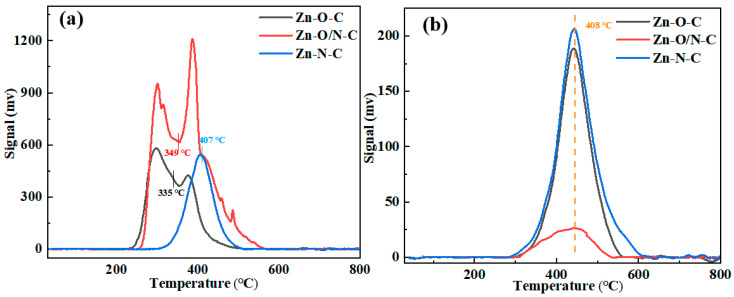
TPD analysis of Zn-O-C, Zn-O/N-C, and Zn-N-C: (**a**) CH_3_COOH and (**b**) C_2_H_2_.

Then, the TPD tests of Zn-O-C, Zn-O/N-C, and Zn-N-C were carried out to observe the adsorption characteristics of the catalysts to the reactants, as shown in Figure 6. The area of the desorption peak represents the adsorption capacity of the reactant, and the desorption temperature indicates the adsorption strength of the reactant. It can be seen that peak temperatures of the catalysts for C_2_H_2_ are basically the same, proving that there is no difference in their adsorption strength for C_2_H_2_. The off-peak areas of Zn-O-C and Zn-N-C are mostly the same, while the off-peak area of Zn-O/N-C is the smallest. Although Zn-O/N-C has the smallest adsorption of C_2_H_2_, the catalytic activity is not very different from Zn-O-C, which shows that the adsorption amount of C_2_H_2_ has little effect on the catalytic activity of the catalysts. The de-peaking temperatures of Zn-O-C, Zn-O/N-C, and Zn-O-C for the catalysts are 335, 349, and 407 °C, respectively, indicating that the Zn-N-C catalyst needs a higher temperature to desorb CH_3_COOH. Comparing the off-peak area, Zn-O/N-C > Zn-O-C > Zn-N-C, it shows that Zn-O/N-C has the largest adsorption capacity for CH_3_COOH, while Zn-N-C has the smallest adsorption capacity for CH_3_COOH; the smaller electron cloud density leads to easier attraction of CH_3_COOH, which corresponds to the position of the Zn 2p binding energy in the XPS characterization [42].

### 3.2. Performance of Catalysts for Acetylene Acetoxylation

**Figure 7:**
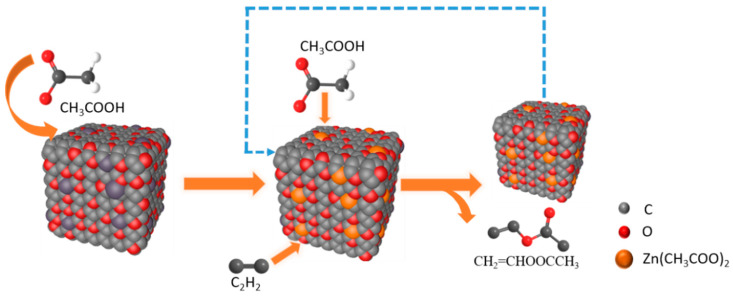
Reaction flow diagram taking Zn-O-C as an example.

At present, there are four mechanisms of catalyzing acetylene acetoxylation, including C_2_H_2_ first absorbed by the catalyst adsorption mechanism, CH_3_COOH first absorbed by the catalyst adsorption mechanism, the acid catalysis mechanism, and the carbonyl promotion mechanism [3]. According to our experiments, we have found that passing C_2_H_2_ in the reaction will deactivate the catalyst. Therefore, we prefer CH_3_COOH first absorbed by the catalyst; the reaction process is shown in Figure 7. First, the active component is combined with CH_3_COOH molecules to form zinc acetate (Figure S4 demonstrates the chemical state of zinc), then C_2_H_2_ molecules are adsorbed for reaction and, finally, CH_3_COOCHCH_2_ is generated after the reaction.

According to the previous analysis, the main factor affecting their activity is the coordination mode of zinc. When the Zn-N bond exists, the electron cloud density around zinc is stronger, which can be proved by the position shift of XPS Zn 2p (Zn 2p binding energy position Zn-N < Zn-O). The increase in the electron cloud density around zinc weakens the attraction for CH_3_COOH molecules, so it is difficult to achieve better catalytic activity. At the same time, it can be found from the TPD spectra that the presence of Zn-N bonds strengthens the binding force of the catalyst and CH_3_COOH. When CH_3_COOH molecules are adsorbed on both sides of the active component at the same time, it is difficult to desorb, which hinders the subsequent reaction. From the TPD diagram of Figure S5, it can be found that Zn-N-C has a larger adsorption capacity for CH_3_COOCHCH_2_ than that of Zn-O-C, which further proves that CH_3_COOH on the Zn-N-C catalyst is difficult to desorb, and it can be inferred that the difficult desorption of CH_3_COOH is the reason for the low activity of the Zn-N-C catalyst.

## 4. Conclusions

In summary, MOFs precursors of MOF5, TEDA-PD, and ZIF8 were prepared by the addition of different organic ligands, resulting in different microscopic morphologies. After the calcination of MOF5, TEDA-PD, and ZIF8, the Zn-O-C, Zn-O/N-C, and Zn-N-C catalysts, respectively, were obtained with the morphologies unchanged. These catalysts were successfully used for acetylene acetoxylation, with the efficiency in the sequence of Zn-O-C (33%), Zn-O/N-C (27%), and Zn-N-C (12%). The characterization analysis revealed that the different organic ligands and the coordination environments resulted in different electron cloud densities around the active components, which further affects the CH_3_COOH adsorption capacity of catalysts and the catalytic reaction activity.

## Data Availability

The data presented in this manuscript are available on request from the corresponding author.

## Supplementary Materials

The following are available online at https://www.mdpi.com/article/10.3390/nano12010098/s1, Figure S1: SEM of each substance 2-MI (a), H_2_BDC (b), H_2_BDC(NH_2_) (c), H_2_BDC(NO_2_) (d), H_2_BDC-TETA-PD (e), H_2_BDC-TEDA-PD (f), H_2_BDC-PD(5) (g), and H_2_BDC-PD(13) (h). (For ease of differentiation, the catalyst in this picture was named after the added ligand.), Figure S2: Reactivity of catalysts with time. (For ease of differentiation, the catalyst in this picture was named after the added ligand.), Figure S3: XPS analysis of the contents of each element (a) and C 1s (b) of Zn-O-C, Zn-O/N-C, and Zn-N-C, Figure S4: XRD patterns in each reaction stage of Zn-O-C catalyst, Figure S5: TPD analysis of Zn-O-C and Zn-N-C catalysts for CH_3_COOCHCH_2_, Table S1: Specific surface area, pore volume, and pore size data of each substance.
