# Supplementary material for: MOFs-Derived Zn-Based Catalysts in Acetylene Acetoxylation
Source: Nanomaterials (Basel). 2021 Dec 29;12(1):98. doi: 10.3390/nano12010098 (PMC8746958; doi:10.3390/nano12010098)
Supplement: Supplementary file 1 [file nanomaterials-12-00098-s001.zip › nanomaterials-1487101 - Supplementary Materials v1.pdf]

nanomaterials

Supplementary Materials

MOFs-Derived Zn-Based Catalysts in Acetylene Acetoxylation

Mengli Li [1], Zhuang Xu [1], Yuhao Chen [1], Guowang Shen [1], Xugen Wang [1,2,*] and Bin Dai [1,2,*]

1 School of Chemistry and Chemical Engineering, Shihezi University, Shihezi 832000, China; 20192007095@stu.shzu.edu.cn (M.L.); 20182007103@stu.shzu.edu.cn (Z.X.); chenyuhao@stu.shzu.edu.cn (Y.C.); 20202107050@stu.shzu.edu.cn (G.S.)
2 Key Laboratory for Green Processing of Chemical Engineering of Xinjiang Bingtuan, Shihezi 832000, China
* Correspondence: wxgen@shzu.edu.cn (X.W.); db_tea@shzu.edu.cn (B.D.); Tel.: +86-993-2057270 (B.D.); Fax: +86-993-2057210 (B.D.)

1. Experimental section

1.1 2-MI-450, 2-MI-550

2-MI-550 was the same sample as Zn-N-C in the text. 2-MI-450 calcination temperature is 450 °C, other steps were the same as the Zn-N-C preparation method.

1.2. H_2BDC-450, $H_2BDC(NH_2)$-450, $H_2BDC(NO_2)$-450, H_2BDC-PD(5)-450, H_2BDC-PD(13)-450

H_2BDC-450 was the same sample as Zn-O-C in the main text. For $H_2BDC(NH_2)$-450, 0.63 g H_2BDC was replaced by 0.4 g H_2BDC and 0.23 g $H_2BDC(NH_2)$, and the other steps were the same as the Zn-O-C preparation method. For $H_2BDC(NO_2)$-450, 0.63 g H_2BDC was replaced by 0.4 g H_2BDC and 0.23 g $H_2BDC(NO_2)$, the other steps were the same as the preparation method of Zn-O-C. H_2BDC-PD(5)-450, H_2BDC-PD(13)-450 was added with 0.5 mL,1.3 mL PD in the addition of $Zn(NO_3)_2 \cdot 6H_2O$ and H_2BDC solution after mixing well, the other steps were the same as the Zn-O-C preparation method.

1.3. H_2BDC-TEDA-PD-450, H_2BDC-TETA-PD-450

H_2BDC-TEDA-PD-450 was the same sample as Zn-O/N-C in the main text. For H_2BDC-TETA-PD-450, TEDA was replaced with TETA, the other steps were the same as the preparation method of Zn-O/N-C.

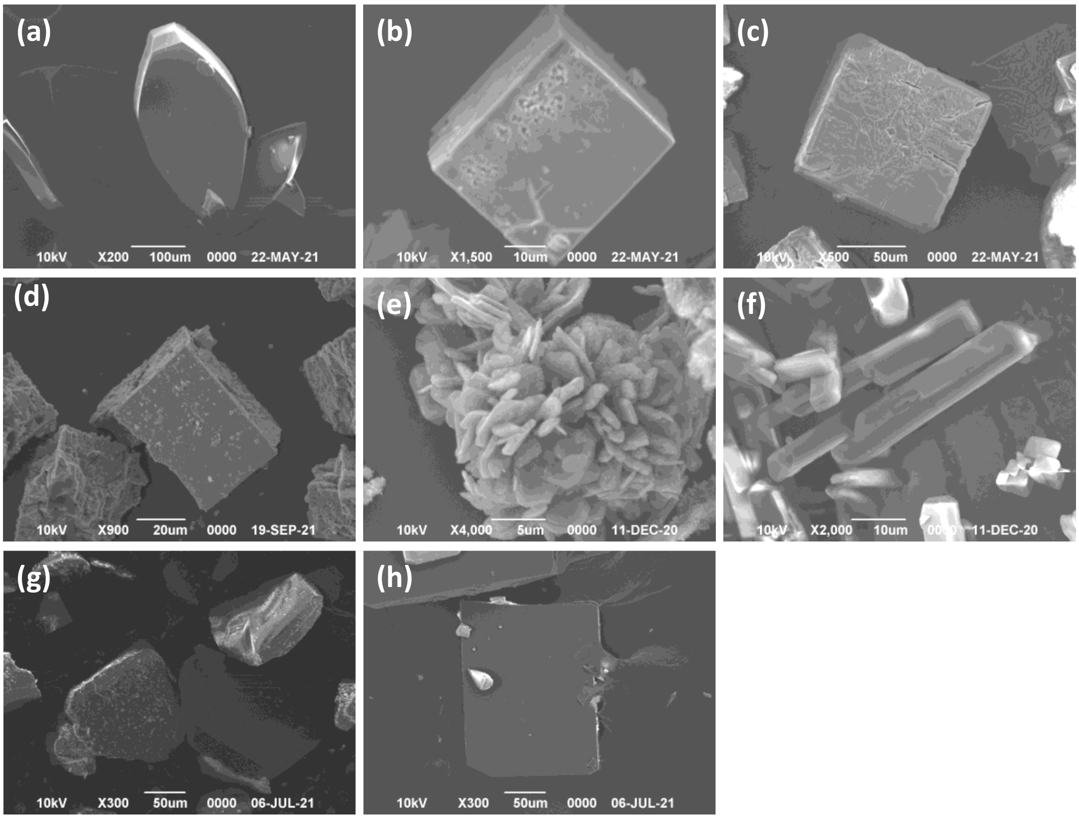

Figure S1. SEM of each substance 2-MI (**a**), H_2BDC (**b**), $H_2BDC(NH_2)$ (**c**), $H_2BDC(NO_2)$ (**d**), H_2BDC-TETA-PD (**e**), H_2BDC-TEDA-PD (**f**), H_2BDC-PD(5) (**g**), H_2BDC-PD(13) (**h**). (For ease of differentiation, the catalyst in this picture was named after the added ligand.)

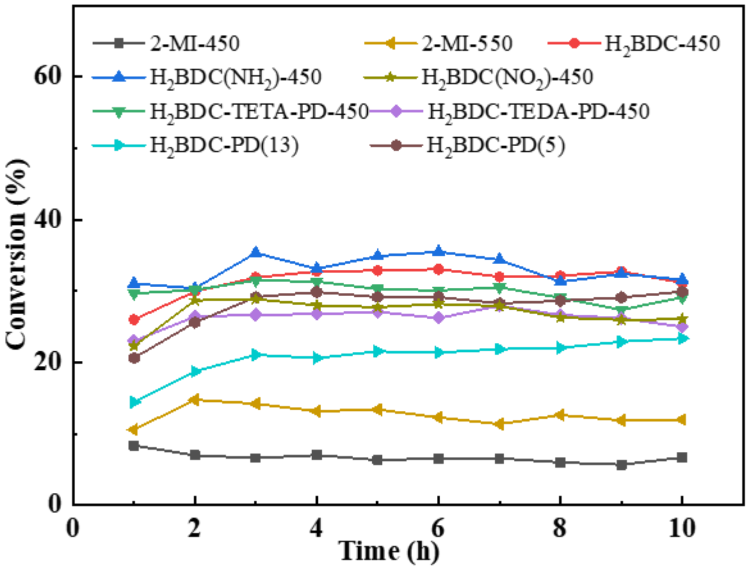

Figure S2. Reactivity of catalysts with time. (For ease of differentiation, the catalyst in this picture was named after the added ligand.)

Table S1. Specific surface area, pore volume, and pore size data of each substance.

Samples	S_{BET} [a] (m^2 g^{-1})	V_{meso} [b] (cm^3 g^{-1})	Dave [c] (nm)
2-MI-450	11.77	0.01	33.17
2-MI-550	331.10	0.10	15.97
H_2BDC-450	301.40	0.33	6.26
H_2BDC(NH_2)-450	338.65	0.58	6.66
H_2BDC(NO_2)-450	426.88	0.59	6.96
H_2BDC-TEDA-PD-450	343.04	0.17	4.91
H_2BDC-TETA-PD-450	291.08	0.20	5.36
H_2BDC-PD(5)-450	345.15	0.26	6.37
H_2BDC-PD(13)-450	297.41	0.14	4.60

(For ease of differentiation, the catalyst in this picture was named after the added ligand.).
[a] The specific surface area (S_{BET}) is obtained by the Brunauer-Emmer-Teller (BET) algorithm;
[b] The mesoporous pore volume (V_{meso}) is calculated by the Barret-Joyner-Halenda (BJH) algorithm to calculate the desorption cumulative volume;
[c] The mesoporous pore size (Dave) is calculated by the Barret-Joyner-Halenda (BJH) algorithm to calculate the desorption average pore width.

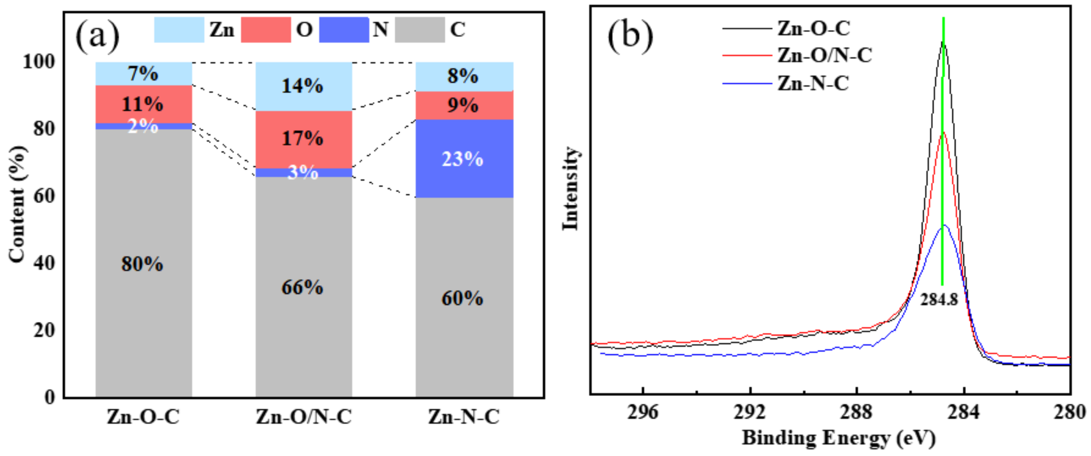

Figure S3. XPS analysis of the contents of each element (**a**) and C 1s (**b**) of Zn-O-C, Zn-O/N-C and Zn-N-C.

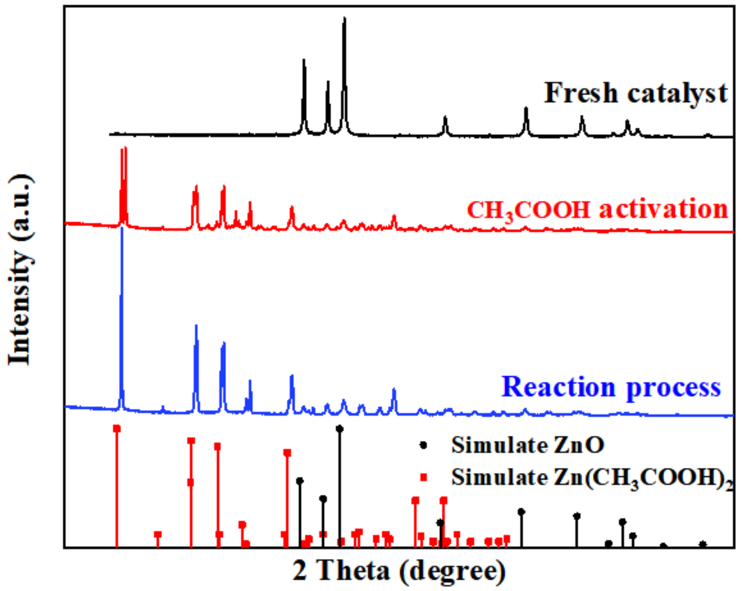

Figure S4. XRD patterns in each reaction stage of Zn-O-C catalyst.

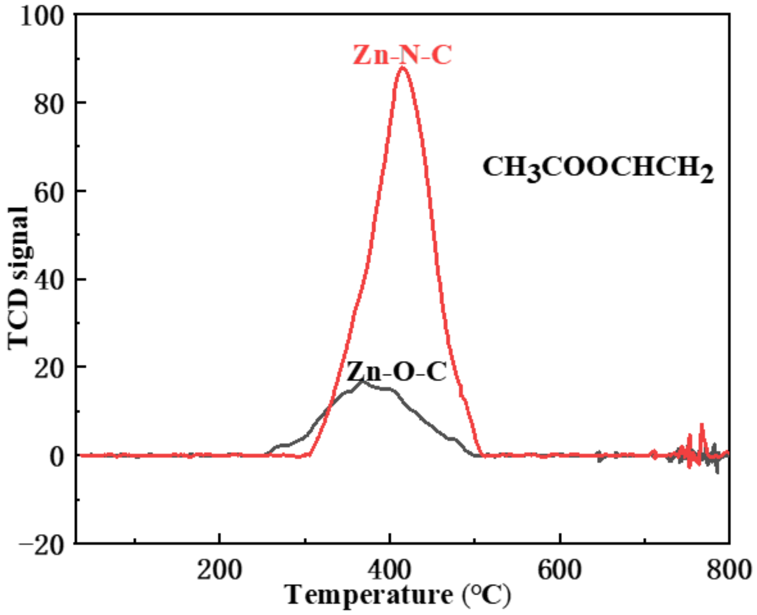

Figure S5. TPD analysis of Zn-O-C and Zn-N-C catalysts for $CH_3COOCHCH_2$.
